# Psychosocial Risk Factors and Musculoskeletal Symptoms among White and Blue-collar Workers at Private and Public Sectors

**DOI:** 10.1186/s40557-014-0020-5

**Published:** 2014-07-25

**Authors:** Leticia B Januario, Mariana V Batistao, Helenice JCG Coury, Ana Beatriz Oliveira, Tatiana O Sato

**Affiliations:** 1 Physical Therapy Department, Physical Therapy Post Graduate Program, Rodovia Washington Luís km 235, São Carlos 13565-905, Brazil

**Keywords:** Cumulative trauma disorders, Ergonomics, Pain threshold, Physical therapy specialty, Prevention and control

## Abstract

**Objectives:**

The aim of this study was to evaluate musculoskeletal and psychosocial perception and compare these conditions regarding the type of job (white or blue-collar) and the type of management model (private or public).

**Methods:**

Forty-seven public white-collar (PuWC), 84 private white-collar (PrWC) and 83 blue-collar workers (PrBC) were evaluated. Job Content Questionnaire (JCQ) and Utrecht Work Engagement Scale (UWES) were applied to evaluate psychosocial factors. Nordic Musculoskeletal Questionnaire (NMQ) was used to assess musculoskeletal symptoms. Pressure Pain Threshold (PPT) was measured to evaluate sensory responses.

**Results:**

According to JCQ, all groups were classified as active profile. There was a significant association between work engagement and workers’ categories (*p* < 0.05). PrWC workers had the highest scores for all the UWES domains, while PrBC had the lowest ones. PPT showed that PrBC workers had an increased sensitivity for left deltoid (*p* < 0.01), and for both epicondyles (*p* < 0.01), when compared to the other groups. PrWC workers had an increased sensitivity for both epicondyles than PuWC (right *p* < 0.01; left, *p* = 0.05). There was no significant association in the report of symptoms across the groups (*p* > 0.05).

**Conclusion:**

This study showed differences in psychosocial risk factors and musculoskeletal symptoms in workers engaged in different types of jobs and work organization. Personal and work-related characteristics, psychosocial factors and PPT responses were different across workers’ group. Despite all, there was no significant difference in reported symptoms across the groups, possibly indicating that the physical load is similar among the sectors.

## Abstract (pt)

**Resumo:**

**Objetivos**

O objetivo deste estudo foi avaliar a percepção musculoesquelética e psicossocial e comparar essas condições em relação ao tipo de trabalho (de escritório ou industrial) e o tipo de modelo de gestão (público ou privado).

**Métodos**

Quarenta e sete trabalhadores de escritório em cargos públicos (PuWC), 84 trabalhadores de escritório em cargos privados (PrWC) e 83 trabalhadores industriais de uma empresa privada (PrBC) foram avaliados. O *Job Content Questionnaire* (JCQ) e a *Utrecht Work Engagement Scale* (UWES) foram aplicados para avaliar os fatores psicossociais. O Questionário Nórdico de Sintomas Osteomusculares (NMQ) foi usado para avaliar os sintomas musculoesqueléticos. O limiar de dor à pressão (PPT) foi medido para avaliar as respostas sensoriais.

**Resultados**

De acordo com JCQ, todos os grupos foram classificados como perfil ativo. Houve uma associação significativa entre o engajamento de trabalho e categorias de trabalhadores (*p* < 0,05). Os trabalhadores PrWC tiveram as maiores pontuações para todos os domínios da UWES, enquanto os PrBC tiveram os menores. Os resultados do PPT mostraram que trabalhadores do grupo PrBC apresentaram maior sensibilidade no deltóide esquerdo (*p* < 0,01) e em ambos os epicôndilos (*p* < 0,01), quando comparados com os outros grupos. Os PrWC apresentaram maior sensibilidade para ambos os epicôndilos do que PuWC (*p* < 0,01 na direita; *p* = 0,05 para esquerda). Não houve associação significativa do relato de sintomas com os grupos (*p* > 0,05).

**Conclusão**

Existe diferença nos fatores de risco psicossociais e sintomas musculoesqueléticos em trabalhadores envolvidos em tipos de trabalho e sistemas organizacionais diferentes. Apesar disso, não houve associação significativa no relato de sintomas entre os grupos, podendo indicar que a demanda biomecânica é semelhante em todos os setores investigados.

## Introduction

Work Related Musculoskeletal Disorders (WRMDs) are one of the most prevalent occupational diseases all over the world. It is responsible for absenteeism, early retirement and disabilities [1]-[5]. According to epidemiologic data provided in 2012 by the Brazilian Social Security Ministry, 720,629 cases of work related accidents and disorders, including WRMDs, were registered in Brazil [6]. Official Brazilian government data do not specify the number of workers who suffered from WRMSDs. The problem is even bigger when considering that almost half of the Brazilian workers are not formally registered in the Social Security Ministry, doing their jobs as informal workers.

Particularly, the neck-shoulder region is exposed to low-level monotonous workload for prolonged periods of time during activities such as office work and industrial repetitive tasks [7],[8].

This overload is associated with many other risk factors, such as high work pace, repetitive and stereotyped movements, maintenance of awkward postures or static seated position, besides temperature, illumination and vibration exposure [7],[9],[10]. The prolonged exposure to these risk factors [11], associated with psychosocial factors [8] and job organization [12], also influences the onset and persistence of WRMDs.

Different levels of exposure are found on workplaces according to the management model and the characteristics of the working activity. When comparing private and public work sectors in Brazil, it is clearly seen that public sector has some particularities, such as: difficulty to promote ergonomics due to inadequate equipment, lack of direct hierarchy, high incidence of strikes, difficulty of career advancement and employment stability [13],[14]. Even though those characteristics are based on the Brazilian reality, this public management model found is similar with other western countries [15],[16]. Bach and Della Rocca [17] reported that the main reason for implementing changes in a Swedish public sector has been to reduce costs instead of promoting better workplaces. Fjell and co-workers [16] have also highlighted another negative issue - the decisions of public authorities can change according to elections outcomes. Therefore, political chiefs can have direct influence on budgets and organizational systems [16].

Biomechanical exposure might be also different among jobs. Even though both white and blue-collar workers perform monotonous and repetitive tasks, each type of job has its particularities. In general, blue-collar workers perform tasks in standing posture, and have more possibilities to adopt other postures, even though the work pace is controlled by the machine or other workers. On the other hand, white-collar workers have more constrained posture, and perform computer-based tasks, requiring seated position and demanding high level of knowledge and attention; one positive feature is the fact that white-collar workers have flexible working rhythm [18],[19].

Thus, the knowledge on risk factors for WRMDs in different jobs, considering management model and working activities is crucial to support preventive actions. In order to contribute with information on this matter, the aim of the present study is to evaluate both musculoskeletal and psychosocial perception among blue-collar and white-collar workers, and compare their condition regarding the type of management model (private or public sector) and the type of job (white-collar or blue-collar).

## Methods

### Description of work sectors and subjects

This cross-sectional study was conducted at a public University and a factory of office supplies, both located at countryside of São Paulo, Brazil. The public university has approximately 888 workers performing administrative tasks. They are distributed among secretaries, and management sectors. They perform office work combined and public attendance. Therefore, their tasks consist of writing e-mails and documents; browsing the web; checking and entering data into spreadsheets; talking on the phone; meeting students or staff; signing documents; moving from one workstation to another to communicate with co-workers and photocopying.

At the factory of office supplies, approximately two thousand workers perform industrial and administrative work. Two sectors were approached in this study. Workers from these sectors are grouped in manufacturing cells, and are responsibly for supplying the machine, supervising the production, inspecting products, and packing. Those tasks involve repetitive manual activities with low levels of muscular contractions and maintenance of awkward postures throughout the workday, characteristics of blue-collar job. The private administrative tasks are similar to the ones described to the university employees.

Two hundred and fourteen workers were recruited to participate in the study. Forty-seven (44.4 ± 8.4 years old) were public white-collar workers (PuWC); 84 (39.0 ± 8.0 years old) were private white-collar workers (PrWC); and 83 (37 ± 8 years old) were private blue-collar workers (PrBC). PuWC were recruited from a list of administrative workers provided by the Human Resource Department. They were randomly contacted by phone calls until a sample of 50 subjects was reached. Three workers, who missed any evaluation, were excluded from the final sample. At the factory, all PrWC and PrBC from two production sectors were invited to participate. Those who participated in all assessments were included in the study.

The study was approved by the Ethics Committee on Human Research of the Federal University of Sao Carlos (Process #352/2010 and #356/2010).

### Instruments and equipment

The evaluation was composed by the application of questionnaires and measurement of the pressure pain threshold. Psychosocial factors were evaluated through the Job Content Questionnaire (JCQ) and the Utrecht Work Engagement Scale (UWES). The JCQ was translated and validated to Brazilian Portuguese [20].

The JCQ correlates the domains of demand and control in order to classify the worker in one of the following domains: active (high demand and high control); passive (low demand and high control); high strain (high demand and low control); low strain (low demand and high control). The domains were classified as high or low according to the median value obtained to each group. The same procedure was applied to classify the social support domain.

The UWES is a questionnaire developed to assess the level of engagement, vigor, dedication and absorption that each subject has in relation with his/her work. Therefore, it requires the worker about the positive aspects of its working activities instead of burnout like most of the available psychosocial questionnaires. There are not current studies validating the UWES to the Brazilian Portuguese, but a translation is provided by the authors [21]. The Cronbach’s α showed good internal consistency for all subitems used on UWES-17 questions (vigor 0.82, dedication 0.89, absorption 0.83, total score 0.93) [21].

The Nordic Musculoskeletal Questionnaire (NMQ) was used to evaluate musculoskeletal symptoms [22]. A Brazilian version of the questionnaire was applied [23]. The pressure pain threshold (PPT) was measured using a mechanical pressure algometer (Pain Diagnosis and Treatment Inc, Great Neck, NY, USA). This device consists of a round rubber disc (1 cm^2^) attached to a pressure gauge, that displays values in kilograms. Both right and left trapezius muscle were tested at half-away between the midline and lateral border of the acromion. The deltoid muscles were tested at the midpoint between the acromion and its insertion [24],[25], and the lateral epicondyles were bilaterally tested [26]. The reference site was located 2 cm below the upper border of the sternum, in the midline.

The subject was evaluated on seated position. The algometer was placed perpendicular to the body surface, at a constant pressure of 1kgf/cm^2^/s. The pressure was interrupted when the subject recognized that the pressure sensation became a pain sensation. Three measurements were performed for each point, with intervals of 30 seconds between them. The mean value, in Kgf, of each point was described as the pain threshold.

### Data analysis

Descriptive analysis including proportion, means, standard deviations and confidence intervals was performed. Data of demographic and personal characteristics were checked for normality and homogeneity of variance through Shapiro Wilks and Levene tests, respectively. Since the assumptions were not attended, the comparison between groups was performed through nonparametric Kruskall-Wallis test. Groups were compared for the categorical dependent variables resulting from the questionnaires (NMQ, JCQ and UWES) by the Chi square association test. Pressure Pain Threshold (PPT) was compared across groups through MANOVA (normality and homogeneity of variance were also checked, and the assumptions were attended). When significant differences were found, univariate tests (one-way ANOVA) identified the significant variables, and Tukey post-hoc test was applied to identify group differences. Logistic regression was applied to identify factors associated with symptoms (age, gender, body mass index, educational level, and JCQ). The stepwise method was used to include variables in the model. The data analysis was performed in SPSS (version 20.0) and the alpha level was set at 5%. There were some missing data; therefore the number of valid cases analyzed is shown in the tables.

## Results

Personal and work-related characteristics of PrWC, PrBC and PuWC, and the total sample are presented in Table 1. Data show that PuWC were older than the workers from both private sectors (white-collar, *p* < 0.01; blue-collar, *p* < 0.01). The private white-collar workers (PrWC) were lighter (*p* = 0.04) and taller (*p* < 0.01) than PuWC. On the other hand, PrWC were also heavier (*p* < 0.01), shorter (*p* < 0.01) and had a greater Body Mass Index - BMI (*p* = 0.01) than PrBC.

**Table 1:** Personal and demographic characteristics for the groups and the total sample

	**PrWC (n = 84)**	**PrBC (n = 83)**	**PuWC (n = 47)**	**Total (n = 214)**	**p-value**
Age - years (mean [SD])	39.1(8.3)	36.9(8.1)	43.4(8.4)	39.4(8.6)	0.000
Weight - kg (mean [SD])	78.6(12.9)	68.3(13.8)	73.6(15.9)	73.5(14.6)	0.000
Height - meters (mean [SD])	1.7(0.1)	1.7 (0.1)	1.6 (0.1)	1.7(0.1)	0.000
BMI - kg/m^2^ (mean [SD])	26.5(3.5)	24.9(4.0)	26.4(5.4)	25.6(4.9)	0.006
Gender (n[%])					
Male	51(60.7)	20(24.1)	9(19.1)	80(37.4)	0.000
Female	33(39.3)	63(75.9)	38(80.9)	134(62.6)	
Scholarity (n[%])					
Incomplete Elementary School	0(0.0)	4(4.8)	0(0.0)	4(1.9)	0.000
Complete Elementary School	0(0.0)	2(2.4)	0(0.0)	2(0.9)	
Incomplete High School	0(0.0)	5(6.0)	1(2.1)	6(2.8)	
Complete High School	3(3.6)	61(73.5)	2(4.3)	66(30.8)	
Technical Education	1(1.2)	7(8.4)	4(8.5)	12(5.6)	
Incomplete University Graduation	11(13.1)	4(4.8)	2(4.3)	17(7.9)	
Complete Graduation	26(31.0)	0(0.0)	10(21.3)	36(16.8)	
Post-graduation	43(51.2)	0(0.0)	28(59.6)	71(3.2)	
Age that started work (mean [SD])	15.5(3.7)	17.2(4.1)	16.7(3.6)	16.(3.9)	0.016
Sickness absence (n[%])	40(47.6)	56(67.5)	16(34.0)	112(52.3)	0.000
Smoke (n[%])	5(6.0)	4(4.8)	2(4.3)	11(5.1)	0.902
Marital status (n[%])					
Single	14(16.7)	17(20.5)	8(17.0)	39(18.2)	0.635
Married	64(76.2)	57(68.7)	32(68.1)	153(71.5)	
Divorced	2(2.4)	5(6.0)	3(6.4)	10(4.7)	
Living with partner	4(4.8)	4(4.8)	3(6.4)	11(5.1)	
Widower	0(0.0)	0(0.0)	1(2.1)	1(0.5)	
Physical Activity	26(31.3)	26(31.3)	20(43.5)	72(34.0)	0.305

The sample size is indicated for each group.

Private white-collar – PrWC, private blue-collar – PrBC, public white-collar – PuWC.

There were more female workers in the public white-collar sector and private blue-collar sector, and more male workers in the private white-collar sector. The majority of PuWC and PrWC have completed post-graduation studies. On the other hand, most of the PrBC have completed high school. The variables marital status (*p* = 0.63), smoke (*p* = 0.90) and physical activity (*p* = 0.30) have not shown association with groups. PrWC have started working earlier than the PrBC (*p* < 0.01) and PuWC (*p* = 0.04). PrBC had higher prevalence of sickness absence than the other workers (*p* < 0.01).

Data of the Utrecht Work Engagement Scale (UWES) are presented in Table 2. Table 3 shows results of the Job Content Questionnaire (JCQ), and Table 4 shows data of the Nordic Musculoskeletal Questionnaire (NMQ) for symptoms reported for the previous 7 days.

**Table 2:** Utrecht Work Engagement Scale (UWES) data from the groups

	**PrWC (n = 84)**		**PrBC (n = 82)**		**PuWC (n = 47)**		**p-value**
	**n (%)**	**95% CI**	**n (%)**	**95% CI**	**n (%)**	**95% CI**	
*Vigor*							
Very low	0(0.0)	0.0-4.4	2(2.9)	0.7-0.9	1(2.1)	0.4-11.1	0.002
Low	1(1.2)	0.2-6.4	14(20.3)	10.5-26.6	6(12.8)	6.0-25.2	
Medium	25(29.8)	21.0-40.2	23(33.3)	19.5-38.6	16(34.0)	22.2-48.3	
High	43(51.2)	40.7-61.6	18(26.1)	14.4-32.1	21(44.7)	31.4-58.8	
Very high	15(17.9)	11.1-27.4	12(17.4)	8.6-23.9	3(6.4)	2.2-17.2	
*Dedication*							
Very low	0(0.0)	0.0-4.4	4(4.9)	1.9-11.9	2(4.3)	1.2-14.2	0.013
Low	6(7.1)	3.3-14.7	15(18.3)	11.4-28.0	8(17.0)	8.9-30.1	
Medium	29(34.5)	25.2-45.2	27(32.9)	23.7-43.7	18(38.3)	25.8-52.6	
High	32(38.1)	28.4-48.4	13(15.9)	9.5-25.3	16(34.0)	22.2-48.3	
Very high	17(20.2)	28.4-48.4	10(12.2)	6.8-21.0	3(6.4)	2.2-17.2	
*Absorption*							
Very low	0(0.0)	0.0-4.4	4(4.9)	1.9-11.9	0(0.0)	0.0-7.6	0.000
Low	2(2.4)	0.7-0.8	16(19.5)	12.4-29.4	4(8.5)	3.4-19.9	
Medium	22(29.8)	18.0-36.5	22(26.8)	18.4-37.3	24(51.0)	37.2-64.7	
High	43(51.2)	40.7-61.6	18(22.0)	14.4-32.1	15(31.9)	20.4-46.2	
Very high	17(20.2)	13.0-30.0	9(11.0)	5.9-19.6	4(8.5)	3.4-19.9	
*Engagement*							
Very low	0(0.0)	0.0-4.4	3(3.7)	1.3-10.2	1(2.1)	0.4-11.1	0.002
Low	2(2.4)	0.7-0.8	14(17.1)	10.5-26.6	6(12.8)	6.0-25.2	
Medium	25(29.8)	21.0-40.2	26(31.7)	22.6-42.4	19(40.4)	27.6-54.7	
High	47(56.0)	45.3-66.1	19(23.2)	15.4-33.4	18(38.3)	25.8-52.6	
Very high	10(11.9)	6.6-20.5	7(8.5)	4.2-16.6	3(6.4)	2.2-17.2	

Number of cases (%). 95% confidence interval (CI 95%). and statistical results (Qui-Square test) are presented.

Private white-collar – PrWC, private blue-collar – PrBC, public white-collar – PuWC.

**Table 3:** Results of the Job Content Questionnaire from private and public workers

	**PrWC (n = 84)**		**PrBC (n = 83)**		**PuWC (n = 47)**		**p-value**
	**n (%)**	**95% CI**	**n (%)**	**95% CI**	**n (%)**	**95% CI**	
Passive	14(16.7)	10.2 - 26.1	4(4.8)	1.9 - 11.7	5(10.6)	4.6 - 22.6	0.046
Low strain	20(23.8)	16.0 - 33.9	22(26.5)	18.2 - 36.9	11(23.4)	13.6 - 37.2	
High strain	21(25.0)	17.0 - 35.2	7(8.4)	4.1 - 16.4	10(21.3)	12.0 - 34.9	
Active	29(34.5)	25.2 - 45.2	37(44.6)	34.4 - 55.3	21(44.7)	31.4 - 58.8	

Number of cases (%). 95% confidence interval (CI 95%). and statistical results (chi square test) are presented.

Private white-collar – PrWC, private blue-collar – PrBC, public white-collar – PuWC.

**Table 4:** Results of the 7 days symptoms for private and public workers

	**PrWC (n = 60)**		**PrBC (n = 63)**		**PuWC (n = 47)**		**p-value**
	**n (%)**	**95% CI**	**n (%)**	**95% CI**	**n (%)**	**95% CI**	
Neck	25(41.7)	30.1 - 54.3	17(27.0)	17.6 - 39.0	13(27.7)	16.9 - 41.8	0.159
Shoulder	20(33.3)	22.7 - 45.9	14(22.2)	13.7 - 33.9	13(27.7)	16.9 - 41.8	0.387
Upper back	16(26.7)	17.1 - 39.0	10(15.9)	8.9 - 26.8	10(21.3)	12.0 - 34.9	0.342
Elbow	5(8.3)	3.6 - 18.1	04(6.3)	2.5 - 15.2	03(6.4)	2.2 - 17.2	0.892
Lower back	17(28.3)	18.5 - 40.8	11(17.7)	10.0 - 28.6	18(38.3)	25.8 - 52.6	0.056
Wrist/Hand	6(10.0)	4.7 - 20.1	7(11.1)	5.5 - 21.2	11(23.4)	13.6 - 37.2	0.098

Number of cases (%), 95% confidence interval (CI 95%), and statistical results (chi square test) are presented.

Private white-collar – PrWC, private blue-collar – PrBC, public white-collar – PuWC.

There was a significant association between the three work groups and the JCQ profiles. All three groups had more workers with the active profile – high demand and high control (PrWC - 34.5%; PrBC – 44.6%; PuWC – 44.7%). The secondly most prevalent profile for PrBC and PuWC was the low strain - low demand and high control (26.5% and 23.4% respectively), while the PrWC were classified as high strain profile - high demand and low control (25%).

Results of the UWES indicate association between workers categories and work engagement (*p* < 0.01), absorption (*p* < 0.01), dedication (*p* = 0.01), and vigor (*p* < 0.01) - Table 3. PrWC had the highest scores for all the domains, while PrBC had the lowest ones.

Both private sectors had high levels of self report symptoms on neck region. However, no association between symptoms report and group was found (*p* = 0.16). Even though this association has not been significant, the *p*-value found when considering the reported pain for the lower back was 0.05. Both white-collar sectors (PuWC and PrWC) had higher prevalence of reported pain in the lower back in the last 7 days than the blue-collar group.

**Figure 1:**
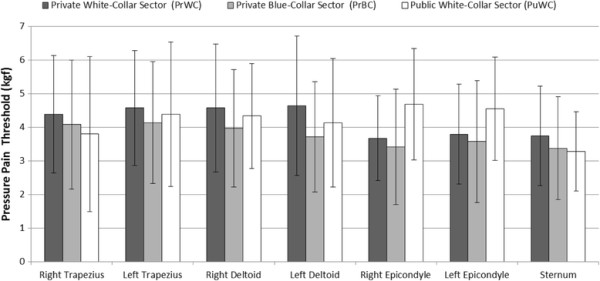
Pressure Pain Threshold (PPT) data for private (white-collar and blue-collar) and public white-collar sector for right and left trapezius, right and left deltoid and right and left lateral epicondyle and sternum.

Data of Pressure Pain Threshold (PPT) are shown in Figure 1. The ANOVA showed no significant difference across groups for the sternum PPT (*p* = 0.18). However, significant differences were found in MANOVA for the other regions (*p* < 0.01). One-way ANOVA showed differences for the left deltoid, right and left epicondyles. For the left deltoid, the PrBC had significant lower pain threshold than the PrWC (Figure 1, *p* < 0.01). For the epicondyles, the PrBC (right and left epicondyle, *p* < 0.01) and PrWC (right epicondyle, *p* < 0.01; left epicondyle, *p* = 0.05) had significant lower pain threshold than the PuWC.

Logistic regression showed JCQ explained 8% of low back symptoms (R^2^ = 0.08; β = 0.34; standard error = 0.16; *p* = 0.03). The odds ratio was 1.4 and the 95% CI was 1.0 to 1.9.

## Discussion

The current study showed differences regarding psychosocial indicators and musculoskeletal symptoms among workers engaged in different types of jobs and work organization. PuWC were older and highly educated than the others groups. PrBC had higher levels of sickness absence. Considering psychosocial factors, all sectors had most of workers classified as active profiles (JCQ) and good work engagement (UWES). Both private sectors had higher prevalence of reported pain on neck region and both white-collar sectors had higher prevalence of reported pain on lower back region according to NMQ. At last, the PPT showed that PrBC had an increased sensitivity for left deltoid and both epicondyles compared to the other workers, and PrWC had an increased sensitivity for both epicondyles compared to PuWC.

According to Marconi [27] a high level of scholarity is required from individuals working at public careers. This requirement occurs during the hiring process for specialized white-collar work tasks. This process allows the subject to be hired on a high job position and get employment stability. However, this leads to a difficulty of career advancement. A study comparing the public and private working population showed that most of public workers are between 41 and 50 years old, and that the job turnover was very low at public administration sectors during 1995 - about 0.4% [27].

The majority of workers evaluated in this study presented active profiles for psychosocial factors according to the demand-control model (JCQ), independently of the sector evaluated. According to Karasek and Theörell [28], the active profile generates a motivational work environment, been considered as a positive profile. However, others studies that evaluated the effects of psychosocial factors on both physical and mental health have found an association between the active and high strain profiles with physical and mental illness, due to the high demand found in both profiles [29]. The worker can respond to increased work demands with a cascade of physiological changes that, if repeatedly evoked, can contributes to the development, exacerbation, and/or maintenance of work-related symptoms [30]. The secondly most frequent profile observed through the JCQ was the low strain, particularly for PrBC and PuWC. The high strain profile was the secondly most prevalent for the PrWC. In this last profile, the low control leads to adverse health effects, like psychological stress, burnout, production of cortisol and adrenalin, self-report irritation and symptoms at the upper limb region [29],[31].

A different and more recent approach to assess psychosocial factors is the evaluation of work engagement. Our results indicated highest work engagement (for both the total score and the three separated dimensions) for private white-collar workers - PrWC, while private blue-collar workers – PrBC, had the lowest work engagement levels compared to the other groups. Despite that, PrBC had work engagement score at medium level, indicating a positive feature. Once the majority of workers were classified as active profile according to JCQ (high control and demand), the good work engagement identified may be consistent with a previous prospective study of the Finnish Public Health Care Personnel. According to previous studies, job control is positively and significantly associated with positive levels of work engagement [32],[33]. This result must be carefully applied to interpret our data since any correlation analysis has been applied. The highest level of work engagement identified among PrWC workers can be partially explained by the job characteristics, since white-collar workers are more engaged than blue-collar workers [21].

These psychosocial indicators may have influenced on the musculoskeletal symptoms, since the PrWC showed greater prevalence (although without statistical significance) of pain in neck, shoulder, upper back and elbow. Regression analysis also reinforces these results, as JCQ explained low back symptoms. Griffiths and co-workers [34] evaluated 934 white-collar workers and have found a significant association between job demands and musculoskeletal symptoms, especially for the neck. However, it is important to consider the ergonomics of the workplace can have an important contribution to the development of musculoskeletal symptoms.

The high prevalence of self-reported pain in all groups (particularly for neck, lower back, shoulders, upper back and wrist/hands) and absence of significant association between groups and pain complaints may suggest that both administrative and blue-collar workers are exposed to potential risk factors for the development of musculoskeletal disorders, independently of the management model. Besides this, confounders variables (age, gender and educational level) were not associated with symptoms. In general, the prevalence of self-reported musculoskeletal symptoms reported in this study agrees with the literature. Nomura et al. [35] evaluated 185 private white-collar workers and observed high levels of self-reported low back pain, and high psychosocial demand through the JCQ. Choobineh et al. [36] evaluated 871 white-collar workers and 313 blue-collar workers of an Iranian petrochemical company. They reported that the most prevalent symptoms among white-collar workers were found in lower back and neck, which agrees with results reported here. On the other hand, the knee was the body region with highest prevalence of symptoms besides the lower back. Griffiths and coworkers [34] have found similar prevalence of self-reported symptoms when evaluating public white-collar workers in Australia.

The body sites with the highest levels of self-reported complaints were both the neck and lower back. This can be explained by the characteristics of the job, such as prolonged exposure to a low-level, monotonous and repetitive workload, associated with awkward postures - mostly in seated position. The neck-shoulder region is specially affected by these characteristics according to the Cinderella hypothesis - submaximal contractions involves a fraction of the motor-units (MUs) available and the recruitment pattern are likely to be stereotyped [37]. Since the recruitment follows the principle of size-ordered MUs, small type I fibers are continuously activated during prolonged and monotonous tasks [7],[8],[38]. The continuous overload on these type I fibers impairs the proper muscle recovery, leading to a harmful process that can cause pain and symptoms.

Considering the lower back region, the literature points out the high prevalence of symptoms among white-collar workers, regardless the management model [37],[39]-[41]. Choobineh et al. [36] compared the self-report of musculoskeletal symptoms of the past 12 months among blue-collar workers, white-collar workers, and workers performing both activities. They reported no differences between groups for complaints on shoulders, elbow, wrists/hands, upper back, lower back, hips/thighs, knees, and ankles/feet. However, they found that workers performing both activities had higher prevalence of symptoms for neck (37.1%) than white-collar (32.5%) and blue-collar workers (24.6%) separately [36].

The evaluation of PPT showed differences between groups for the left deltoid and both right and left epicondyles. PPT measurements are relevant in working populations with musculoskeletal disorders [15],[42]-[46]. PrBC had lower PPT when compared with PrWC (left deltoid) and PuWC (right and left epicondyles). These workers had also a significant higher sickness absence report than the other workers. We have not had access to information about the reason of the sickness absence, difficulting the interpretation of the results. However, a study investigating the work-related sickness absence in United Kingdom found lower prevalence for manufacturing sectors (51%) and higher prevalence for public administrative sectors (71%) than the ones found in our study [16].

Differences between PrWC and PuWC were also found. PrWC had a significant lower PPT for both right and left epicondyles than PuWC. Private white-collar workers have also presented higher prevalence of sickness absence than the white-collar workers from the public sector. Binderup and coworkers [35] found similar results when evaluating 29 cleaners. They found a relationship between long-term sickness absence and a lower level of PPTs in the neck-shoulder region.

Lower values of PPTs have been associated with frequency of forearm and shoulder symptoms [41]. Nielsen and coworkers [43] evaluated 70 female white-collar workers and found that participants with trapezius myalgia had a lower PPT, when compared with healthy controls. Binderup and coworkers [42] found a negative correlation between the mean PPTs of the cervico-thoracic region and the self-reported pain in neck, dominant shoulder and upper back within the last 7 days among cleaners. In our study, the PrWC group presented low PPT on the epicondyles combined to a higher prevalence of elbow symptoms. On the other hand, this behavior was not seen among PrBC. Private blue-collar workers presented low PPT on both deltoid and epicondyles and a relatively low prevalence of symptoms on those regions. However, we may not discard the hypotheses of these workers being on an initial phase of some musculoskeletal disorder. In a longitudinal study of initially asymptomatic blue-collar workers, Madeleine and coworkers [44] found that low PPTs are already seen in those workers who developed musculoskeletal symptoms 6 months later, reflecting the cumulative and chronic nature of the WRMDs.

This study has some limitations such as: the cross-sectional design limits the cause-effect understanding and the lack of biomechanical exposure measurements. Despite that, the results contribute to improve knowledge on this field of expertise, allowing to understand the behavior of psychosocial factors, musculoskeletal symptoms and sensory responses across sectors with different organizational aspects but exposed to a monotonous and repetitive workload.

The present study showed differences in psychosocial indicators and musculoskeletal symptoms in workers engaged in different types of jobs and work organization. Personal and work-related characteristics, psychosocial factors and PPT responses were different across workers’ group. The majority of workers have a high psychosocial load, particularly the private white-collar workers. Despite this, all sectors showed a good work engagement, although the results of the private white-collar group have been better than the other ones.

The blue-collar workers had an increased sensitivity, by means of the PPT, for left deltoid and both epicondyles compared to the other workers. Moreover, the private white-collar workers had an increased sensitivity for both epicondyles compared to the public ones. The low PPT may have reflected on the sickness absence of the white-collar and blue-collar private workers, and on musculoskeletal symptoms only for the private white-collar workers. Despite all, there was no significant difference in reported symptoms across the groups, possibly indicating that the physical load is overall similar among the sectors.

## Abbreviations

ANOVA: Analysis of variance

cm: centimeter

JCQ: Job Content questionnaire

kgf: kilogram-force

MANOVA: Multivariate analysis of variance

MU: Motor unit

NMQ: Nordic musculoskeletal questionnaire

PrBC: Private blue-collar workers

PrWC: Private white-collar workers

PPT: Pressure pain threshold

PuWC: Public white-collar workers

s: seconds

UWES: Utrecht work engagement scale

## Competing interests

The authors declare that they have no competing interests.

## Authors’ contributions

All authors made substantive intellectual contributions to this study to qualify as authors. LBJ, HJCGC, ABO and TOS participated in the design, approval, and collection. LBJ, HJCGC, ABO, MVB and TOS participated in the analysis, interpretation of data and writing of this study. All authors reviewed the final version, gave the final approval of the version to be published, and agreed to be accountable for all aspects of the work in order to ensure that questions related to the accuracy or integrity of any part of the work will be appropriately investigated and resolved.

## References

[B1] David G Woods V Li G Buckle P The development of the Quick Exposure Check (QEC) for assessing exposure to risk factors for work-related musculoskeletal disorders Appl Ergon 2008 39 57 69 10.1016/j.apergo.2007.03.002 17512492

[B2] Bevan S Quadrello T McGee R Mahdon M Vovrovsky A Barham L Fit For Work - Musculoskeletal disorders in the European workforce 2009 The Work Foundation, London

[B3] Bergstrom G Bodin L Bertilsson H Jensen IB Risk factors for new episodes of sick leave due to neck or back pain in a working population. A prospective study with an 18-month and a three-year follow-up Occup Environ Med 2007 64 279 287 10.1136/oem.2006.026583 17095548 PMC2078447

[B4] Nyman T Grooten WJ Wiktorin C Liwing J Norrman L Sickness absence and concurrent low back and neck-shoulder pain: results from the MUSIC-Norrtälje study Eur Spine J 2007 16 631 638 10.1007/s00586-006-0152-6 16741741 PMC2213552

[B5] Lund T Labriola M Christensen KB Bultmann U Villadsen E Physical work environment risk factors for long term sickness absence: prospective findings among a cohort of 5357 employees in Denmark BMJ 2006 332 449 452 10.1136/bmj.38731.622975.3A 16446280 PMC1382535

[B6] *Ministério da Previdência Social, Instituto Nacional do Seguro Social, Empresa de Tecnologia e Informações da Previdência Social: Anuário Estatístico da Previdência Social 2012.* Brasília: Secretaria de Políticas de Previdência Social; 2012.

[B7] Punnett L, Bergqvist U: **Visual display unit work and upper extremity musculoskeletal disorders. A review of epidemiological findings. (National Institute for Working Life - Ergonomic Expert Committee Document No 1).** *Arbete Och Hälsa* 1997, 1–161.

[B8] Cote P Velde G Cassidy JD Carroll LJ Hogg-Johnson S Holm LW Carragee EJ Haldeman S Nordin M Hurwitz EL Guzman J Peloso PM The burden anddeterminants of neck pain in workers – results of the bone and joint decade 2000–2010 task force on neck pain and its associated disorders J Spine 2008 33 60 74 10.1097/BRS.0b013e3181643ee4

[B9] Bernard B Musculoskeletal Disorders and Workplace Factors: A Critical Review of Epidemiologic Evidence for Work-Related Musculoskeletal Disorders of the Neck, Upper Extremity, and Low Back (DHHS/NIOSH publication no.97-141) 1997 Government Printing White-collar, U.S. Washington

[B10] Haynes S Williams K Impact of seating posture on user comfort and typing performance for people with chronic low back pain Int J Ind Ergon 2008 38 35 46 10.1016/j.ergon.2007.08.003

[B11] Lassen CF Mikkelsen S Kryger AI Andersen JH Risk factors for persistent elbow, forearm and hand pain among computer workers Scand J Work Environ Health 2005 31 122 131 10.5271/sjweh.859 15864906

[B12] Westgaard RH Winkel J Occupational musculoskeletal and mental health: significance of rationalization and opportunities to create sustainable production systems – a systematic review Appl Ergon 2011 42 261 296 10.1016/j.apergo.2010.07.002 20850109

[B13] Filho JMJJ Work design and “sick workplace syndrome”. A case study in a public institution Revista Produção 2004 14 58 66

[B14] Oliveira AB Vingård E Gil Coury HJC Sintomas musculoesqueléticos e fatores de risco físicos e psicossociais entre funcionários administrativos do setor público 2008 Anais do XV Congresso Brasileiro de Ergonomia - ABERGO, Porto Seguro, Bahia

[B15] Ciccarelli M Straker L Mathiassen SE Pollock C Diversity of tasks and information technologies used by office workers at and away from work Ergonomics 2011 54 11 1017 1028 10.1080/00140139.2011.609913 22026945

[B16] Fjell Y Österberg M Alexanderson K Karlqvist L Bildt K Appraised leadership styles, psychosocial work factors, and musculoskeletal pain among public employees Int Arch Occup Environ Health 2007 81 19 30 10.1007/s00420-007-0189-9 17415585

[B17] Bach S Della Rocca G The management strategies of public service employers in Europe Ind Relat J 2000 31 82 96

[B18] Vandelanotte C Duncan MJ Short C Rockloff M Ronan K Happell B Milia LD Associations between occupational indicators and total, work-based and leisure-time sitting: a cross-sectional study BMC Public Health 2013 13 1110 10.1186/1471-2458-13-1110 24289321 PMC3879072

[B19] Schreuder KJ Roelen CA Koopmans PC Groothoff JW Job demands and health complaints in white and blue collar workers Work 2008 31 4 425 432 19127013

[B20] Alves MGM Chon D Faerstein E Lopes CS Werneck GL Short version of the “job stress scale”: a Portuguese-language adaptation Rev Saude Publica 2004 38 164 171 10.1590/S0034-89102004000200003 15122370

[B21] Schaufeli W Bakker A UWES Utrecht Work Engagement Scale. Preliminary Manual [Version 1.1] 2004 Utrecht University - Occupational Health Psychology Unit, Utrecht

[B22] Kuorinka I Jonsson B Kilbom A Vinterberg H Biering-Sørensen F Andersson G Jørgensen K Standardized Nordic questionnaire for the analysis of musculoskeletal symptoms Appl Ergon 1987 18 233 237 10.1016/0003-6870(87)90010-X 15676628

[B23] Pinheiro FA Tróccolia BT Carvalho CV Validação do Questionário Nórdico de Sintomas Osteomusculares como medida de morbidade Rev Saude Publica 2002 36 307 312 10.1590/S0034-89102002000300008 12131969

[B24] Jones D Kilgour RD Comtois AS Test-retest reliability of pressure pain threshold measurements of the upper limb and torso in young healthy women J Pain 2007 8 650 656 10.1016/j.jpain.2007.04.003 17553750

[B25] Ylinen J Nyka M Kautiainenc H Hakkinena A Evaluation of repeatability of pressure algometry on the neck muscles for clinical use Man Ther 2007 12 192 197 10.1016/j.math.2006.06.010 16956783

[B26] Pienimäki TT Siira PT Vanharanta H Chronic medial and lateral epicondylitis: a comparison of pain, disability, and function Arch Phys Med Rehabil 2002 83 317 321 10.1053/apmr.2002.29620 11887110

[B27] Marconi N Uma breve comparação entre os mercados de trabalho do setor público e privado Rev Serviço Público 1997 48 126 146

[B28] Karasek RA Theörell T Healthy work-stress, productivity, and the reconstruction of working life 1990 Basic Books, New York

[B29] Araújo TM Graça CC Araújo E Estresse ocupacional e saúde: contribuições do Modelo Demanda-Controle Cien Saude Colet 2003 8 991 1003 10.1590/S1413-81232003000400021

[B30] Feuerstein M Biobehavioral mechanisms of work-related upper extremity disorders: a new agenda for research and practice Am J Ind Med 2002 41 293 297 10.1002/ajim.10060 12071485

[B31] Frankenhaeuser M A biopsychosocial approach to work life issues The psychosocial work environment work organization, democratization and health – essays in memory of Bertil Gardell 1991 Baywood Publishing Company, New York 49 60

[B32] Nerstad CGL Richardsen AM Martinussen M Factorial validity of the Utrecht Work Engagement Scale (UWES) acrossoccupational groups in Norway Scand J Psychol 2010 51 326 333 20015117 10.1111/j.1467-9450.2009.00770.x

[B33] Mauno S Kinnunen U Ruokolainen M Job demands and resources as antecedents of work engagement: a longitudinal study J Vocat Behav 2007 70 149 171 10.1016/j.jvb.2006.09.002

[B34] Griffiths KL Mackey MG Adamson BJ Behavioral and psychophysiological responses to job demandsand association with musculoskeletal symptoms in computer work J Occup Rehabil 2011 21 482 492 10.1007/s10926-010-9263-3 21327727

[B35] Nomura K Nakao M Sato M Ishikawa H Yano E The association of the reporting of somatic symptoms with job stress and active coping among japanese White-collar workers J Occup Health 2007 49 370 375 10.1539/joh.49.370 17951968

[B36] Choobineh A Sani GP Rohani MS Pour MG Neghab M Perceived demands and musculoskeletal symptoms among employees of an Iranian petrochemical industry Int J Ind Ergon 2009 39 766 770 10.1016/j.ergon.2009.01.001

[B37] Visser B Van D Pathophysiology of upper extremity muscle disorders J Electromyogr Kinesiol 2006 16 1 16 10.1016/j.jelekin.2005.06.005 16099676

[B38] Hägg G Anderson PA, Hobart DJ, Danoff JV Static workloads and occupational myalgia – a new explanation model Electromyographical kinesiology 1991 Elsevier Science Publishers, Amsterdam 141 144

[B39] Hemingway H Shipley MJ Stansfeld S Marmot M Sickness absence from back pain, psychosocial work characteristics and employment grade among white-collar workers Scand J Work Environ Health 1997 23 121 129 10.5271/sjweh.189 9167235

[B40] Juul-Kristensen B Jensen C Self-reported workplace related ergonomic conditions as prognostic factors for musculoskeletal symptoms: the “BIT” follow up study on white-collar workers Occup Environ Med 2005 62 188 194 10.1136/oem.2004.013920 15723884 PMC1740969

[B41] Juul-Kristensen B Søgaard K Strøyer J Jensen C Computer users’ risk factors for developing shoulder, elbow and back symptoms Scand J Work Environ Health 2004 30 390 398 10.5271/sjweh.827 15529802

[B42] Binderup AT Holtermann A Søgaard K Madeleine P Pressure pain sensitivity maps, self-reported musculoskeletaldisorders and sickness absence among cleaners Int Arch Occup Environ Health 2011 84 647 654 10.1007/s00420-011-0627-6 21400102

[B43] Nielsen PK Andersen LL Olsen HB Rosendal L Sjøgaard G Søgaard K Effect of physical training on pain sensitivity and trapezius muscle morphology Muscle Nerve 2010 41 836 844 10.1002/mus.21577 20513105

[B44] Madeleine P Voigt M Arendt-Nielsen L Sensory manifestations in experimental and work-related chronic neck-shoulder pain Eur J Pain 1998 2 251 260 10.1016/S1090-3801(98)90021-0 15102385

[B45] Madeleine P Lundager B Voigt M Arendt-Nielsen L The effects of neck–shoulder pain development on sensory–motorinteractions among female workers in the poultry and fish industries - a prospective study Int Arch Occup Environ Health 2003 76 39 49 12592581 10.1007/s00420-002-0375-8

[B46] Hägg GM Åström A Load pattern and pressure pain threshold in the upper trapezius muscle and psychosocial factors in medical secretaries with and without shoulder/neck disorders Int Arch Occup Environ Health 1997 69 423 432 10.1007/s004200050170 9215929

